# Coronary artery disease risk factors affected by RNA modification-related genetic variants

**DOI:** 10.3389/fcvm.2022.985121

**Published:** 2022-09-20

**Authors:** Ru Li, Huan Zhang, Fan Tang, Chengcheng Duan, Dan Liu, Naqiong Wu, Yonghong Zhang, Laiyuan Wang, Xingbo Mo

**Affiliations:** ^1^Jiangsu Key Laboratory of Preventive and Translational Medicine for Geriatric Diseases, Department of Epidemiology, School of Public Health, Soochow University, Suzhou, China; ^2^Key Laboratory of Cardiovascular Epidemiology, State Key Laboratory of Cardiovascular Disease, Department of Epidemiology, National Center for Cardiovascular Diseases, Fuwai Hospital, Chinese Academy of Medical Sciences and Peking Union Medical College, Beijing, China; ^3^Cardiometabolic Center, National Center for Cardiovascular Diseases, Fuwai Hospital, Chinese Academy of Medical Sciences and Peking Union Medical College, Beijing, China; ^4^Center for Genetic Epidemiology and Genomics, School of Public Health, Soochow University, Suzhou, China

**Keywords:** RNA modification, genome-wide association study, gene expression, cardiotrophin-1, Mendelian randomization

## Abstract

**Background:**

Single nucleotide polymorphisms that affect RNA modification (RNAm-SNPs) may have functional roles in coronary artery disease (CAD). The aim of this study was to identify RNAm-SNPs in CAD susceptibility loci and highlight potential risk factors.

**Methods:**

CAD-associated RNAm-SNPs were identified in the CARDIoGRAMplusC4D and UK Biobank genome-wide association studies. Gene expression and circulating protein levels affected by the RNAm-SNPs were identified by QTL analyses. Cell experiments and Mendelian randomization (MR) methods were applied to test whether the gene expression levels were associated with CAD.

**Results:**

We identified 81 RNAm-SNPs that were associated with CAD or acute myocardial infarction (AMI), including m^6^A-, m^1^A-, m^5^C-, A-to-I- and m^7^G-related SNPs. The m^6^A-SNPs rs3739998 in *JCAD*, rs148172130 in *RPL14* and rs12190287 in *TCF21* and the m^7^G-SNP rs186643756 in *PVT1* were genome-wide significant. The RNAm-SNPs were associated with gene expression (e.g., *MRAS, DHX36, TCF21, JCAD* and *SH2B3*), and the expression levels were associated with CAD. Differential m^6^A methylation and differential expression in FTO-overexpressing human aorta smooth muscle cells and peripheral blood mononuclear cells of CAD patients and controls were detected. The RNAm-SNPs were associated with circulating levels of proteins with specific biological functions, such as blood coagulation, and the proteins (e.g., cardiotrophin-1) were confirmed to be associated with CAD and AMI in MR analyses.

**Conclusion:**

The present study identified RNAm-SNPs in CAD susceptibility genes, gene expression and circulating proteins as risk factors for CAD and suggested that RNA modification may play a role in the pathogenesis of CAD.

## Introduction

Coronary artery disease (CAD) is the leading global cause of death (1). As an archetypal common complex disease, a person's risk of developing CAD is modulated by a combination of genetic and environmental factors (2). Family-based and twin-pair studies showed that CAD is partly heritable due to genetic and possibly epigenetic factors, and its broad-sense heritability has been estimated to be between 40 and 60% (3). Over the past decade, great progress has been made in understanding the genetics of CAD (4). Genome-wide association studies (GWASs) have provided valuable clues to the understanding of the pathophysiology of this complex disease. More than 100 CAD-associated loci have been identified by previous large-scale GWAS (5–11). Some genetic variants in the identified loci have been shown to affect gene expression. However, most of their functions remain largely unknown.

Since the first discovery of modifiable nucleosides, more than 170 distinct RNA modification types have been detected in all types of RNA molecules (12, 13). Modifiable RNA modifications are involved in the regulation of various biological processes in eukaryotic cells (14). In the study of posttranscriptional gene regulation, RNA modifications are becoming increasingly important. Several RNA modification types, including m^6^A (N6-adenosine methylation), m^6^Am (N6,2'-O-dimethyladenosine), m^7^G (N7-methylguanosine), m^1^A (N1-adenosine methylation), m^5^C (5-methylcytidin), m^5^U (5-methyluridine), Nm (ribose 2'-O-methylation), pseudouridine and A-to-I RNA editing, have been explored since sufficiently sensitive high-resolution transcriptome-wide techniques are available. N6-methyladenosine (m^6^A) is the most common reversible RNA methylation that plays roles in nearly every aspect of the mRNA lifecycle and in various disease processes (15). m^6^A methylation has become a research hotspot. Recently, genetic variants, e.g., RNA modification-related SNPs (RNAm-SNPs), that impact several types of RNA modifications by changing the modification target sites or RNA sequences around modifiable nucleotides have been highlighted (16). The RNAm-SNPs in CAD-associated genomic loci may have impacts on RNA modifications and may be putative functional variants for CAD. However, studies on the relationship between RNAm-SNPs and CAD are very scarce.

The identification of RNAm-SNPs as functional variants should help with the translation of GWAS signals into causal mechanisms and clinical applications. The aim of this study was to evaluate the impacts of RNAm-SNPs on CAD using publicly available GWAS summary datasets. Quantitative trait locus (QTL) analysis, cell experiments and Mendelian randomization (MR) analysis were performed to identify the role of the RNAm-SNPs.

## Methods

### Determination of RNAm-SNPs for CAD

The RNAm-SNPs annotation information was obtained in the RMVar database (http://rmvar.renlab.org/download.html), which contains 1,678,126 RNAm-SNPs related to the nine kinds of RNA modifications (m^6^A, m^5^C, m^5^U, m^7^G, m^1^A, m^6^Am, 2′-O-Me, A-to-I and pseudouridine) (17). In the RMVar database, the RNAm-SNPs are classified into three categories according to confidence levels: high, medium and low. RNAm-SNPs determined by single-nucleotide resolution experiments (e.g., miCLIP, m6A-Label-seq, BS-Seq and DART-seq) have high confidence. RNAm-SNPs obtained from MeRIP-Seq experiments were defined as having a medium confidence level. m^6^A sites with low confidence levels were predicted by a statistical model.

We obtained functional interpretations for CAD GWAS signals by integrating novel RNA modification annotations (i.e., RNAm-SNPs) and summary data from the CARDIoGRAMplusC4D CAD GWAS (7). The GWAS examined the associations between genome-wide SNPs and CAD risk in approximately 185 thousand individuals, mainly (77%) of European descent. The raw data used in our analysis were summary statistics downloaded at http://www.cardiogramplusc4d.org/data-downloads/. The dataset embodied effect size (beta), standard error and *P* values of associations between approximately 9.5 million SNPs and CAD.

We also annotated RNAm-SNPs in UK Biobank GWAS summary data of acute myocardial infarction (AMI, ICD-10 code: I21). The UK Biobank AMI GWAS included 8,764 AMI patients and 443,500 controls (18). GWAS summary data of AMI (ICD-10 code: I21) were obtained at http://geneatlas.roslin.ed.ac.uk/downloads/. The dataset comprises summary statistics of the genome-wide associations between 9,113,133 imputed variants and AMI analyzed in the linear mixed models. Detailed information on the GWAS can be identified in previous UK Biobank publications (19).

When the GWAS summary data files were downloaded, the CAD-associated SNPs were selected (*P* < 1.0 × 10^−4^ were considered). Then, the dataset containing the CAD-associated SNPs was combined with the RNAm-SNP annotation files based on the “rs ID numbers” column using the “merge” function of the R program. By this combination, the SNPs in GWAS summary datasets were annotated according to the RNA modification annotations of the RNAm-SNP sets. Then, RNAm-SNPs associated with CAD were identified.

### eQTL analysis for the RNAm-SNPs

RNA modification plays a critical role in gene expression regulation. Therefore, CAD-associated RNAm-SNPs may influence gene expression by affecting RNA modification. We performed gene expression QTL (eQTL) analysis to further elaborate the association between the CAD-associated RNAm-SNPs and mRNA expression levels in several types of tissues. We carried out *cis*-acting eQTL analysis in different cells and tissues to obtain functional evidence for the RNAm-SNPs by searching data from the HaploReg browser (http://archive.broadinstitute.org/mammals/haploreg/haploreg.php) (20). Signals in 10 relevant tissues (coronary artery, aortic artery, tibial artery, atrial appendage, left ventricle, liver, visceral omentum adipose, subcutaneous adipose, adrenal gland and pancreas) and whole blood cells were considered.

### SMR analysis

We performed a summary data–based Mendelian randomization (SMR) (21) analysis to identify associations between gene expression levels and CAD by integrating eQTL data from the GTEx project (22) with the CARDIoGRAMplusC4D GWAS data. The eQTL datasets that contain summary statistics of gene expression levels in 10 relevant tissues (coronary artery, aorta artery, tibial artery, atrial appendage, left ventricle, liver, visceral omentum adipose, subcutaneous adipose, adrenal gland and pancreas) and whole blood cells were used in the analysis (http://cnsgenomics.com/software/smr/#DataResource). The CAD summary statistics required in the SMR analysis were sorted from the CARDIoGRAMplusC4D GWAS datasets. In SMR analysis, the top-associated eQTL for each gene was used as an instrumental variable to examine association with CAD.

SMR software (version 0.712) was used with default parameters. Genotype data from HapMap r23 CEU were used as a reference panel to calculate the correlation matrix for SNPs analyzed in SMR. The significance threshold was adjusted with Bonferroni correction. The genome-wide significance threshold for the SMR analysis was 5.0 × 10^−5^. Meanwhile, the heterogeneity in dependent instruments (HEIDI) test was performed to test the ‘no horizontal pleiotropy' assumption, the basic assumption of MR study. The HEIDI test was conducted to examine whether there was a single causal SNP affecting CAD and gene expression. Multiple testing correction is not required in the HEIDI test, as Zhu et al. did in the original paper of this method (21). Genes without heterogeneity (*P*_HEIDI_ ≥ 0.05) were considered. The results of the HEIDI tests are presented in the Supplementary Tables S1–S6.

### Cell culture and transfection

We attempted to determine whether the interference of RNAm-SNPs on m^6^A methylation and gene expression affects CAD. FTO is an RNA demethylase whose major substrate is m^6^A methylation and is an important regulator of mRNA expression. Overexpression of FTO decreased m^6^A levels on RNA (23). We therefore compared the effect of FTO overexpression on m^6^A modification and the expression of the genes identified to be affected by the RNAm-SNPs in human aorta smooth muscle cells (HASMCs). The HASMCs were purchased from ScienCell (Catalog #6110, San Diego, CA, USA). On receiving them, cells were seeded into each well of 6-well plates at a concentration of 2 × 10^6^ cells per plate and cultured in Smooth Muscle Cell Medium supplemented with 2% fetal bovine serum (Catalog #0010; Thermo Fisher Scientific), 1% smooth muscle cell growth supplement (Catalog #1152) and 1% penicillin/streptomycin solution (Catalog #0503). Cells were grown at 37°C in a humidified 5% CO_2_ incubator. The medium was replaced every 48 h. When cells were cultured to subconfluence, they were subcultured with 0.25% trypsin-EDTA (Gibco, Life Technology, USA) at a ratio of 1:3. HASMCs from passages 5 to 7 were used in the following experiments. For adenovirus-mediated overexpression, Ad-FTO (Vigenebio, Shandong, China) was added to the culture medium, and HASMCs were transfected with Ad-FTO at a multiplicity of infection of 100 at 37°C. The transfected HASMCs were incubated for an additional 48 h. A recombinant adenovirus encoding enhanced GFP (Ad-GFP) was used as a negative control.

### MeRIP-seq and RNA-seq

RNA was extracted from peripheral blood mononuclear cells (PBMCs) of five CAD patients and five healthy controls (24) and from FTO-overexpressing HASMCs (*n* = 3) and control HASMCs (*n* = 3) for MeRIP-seq and RNA-seq. The MeRIP-seq experiment was conducted by Guangzhou Epibiotek Co., Ltd. (Guangzhou, China) in compliance with our previously published procedure (24) with slight modifications. Briefly, RNAs were fragmented into 100 nt fragments after removing rRNA with a Ribo-Zero rRNA Removal Kit (Illumina, MRZG12324). A strand-specific RNA library was constructed using 10 ng fragmented RNA in accordance with the UTP method. The remaining fragmented RNA was incubated with anti-m^6^A polyclonal antibody (Synaptic Systems, 202003) in immunoprecipitation (IP) buffer at 4°C for 2 h. The mixture was then immunoprecipitated with protein-A beads (Thermo Fisher Scientific) at 4°C for an additional 2 h. Then, the immunoprecipitated RNA was eluted from the beads with N6-methyladenosine (Berry & Associates, PR3732) in IP buffer and extracted with TRIzol reagent (Thermo Fisher Scientific, 15596026). Purified RNA (the m^6^A IP and the input samples without IP sample each) was used for RNA-seq library construction with the NEBNext^®^ Ultra™ II Directional RNA Library Prep Kit for Illumina^®^ (NEB, #E7760). The IP library (MeRIP-seq) and input library (RNA-seq) were each subjected to an Illumina HiSeq 4000 sequencer (Illumina, Inc.) with 150 bp paired-end reads. The harvested paired-end reads were analyzed by Q30. When the Q30 > 80%, the reads were qualified. The clean reads of all libraries were obtained after trimming 3' adaptors, and low-quality reads were removed by Cutadapt software (v1.9.3) (25); then, the reads were aligned by using Hisat2 software (v2.0.4) (26). MACS software (27) was used to identify methylated peaks on RNAs. DiffReps software (28) was used to identify differentially methylated lncRNAs/mRNAs.

### Differential expression analysis

We further examined the differential expression of the identified genes in PBMCs. Transcriptome-wide lncRNA and mRNA expression profiles of PBMCs of 93 CAD patients and 48 healthy controls were obtained. Details of our transcriptome-wide lncRNA and mRNA expression profile were described in a previous publication (29). The microarray data were normalized and analyzed using GeneSpring software V12.0 (Agilent Technologies). Differential expression of the identified CAD-related genes between CAD cases and controls was tested by comparing mean gene expression levels between cases and controls using *t* tests. The *P* value was two-tailed. Probes with fold change > 2.0 and FDR <0.05 were considered differentially expressed.

### pQTL analysis for the CAD-associated RNAm-SNPs

To look for proteins affected by the RNAm-SNPs, we carried out pQTL analysis in peripheral blood by using data from a published pQTL study, the INTERVAL study. This pQTL study tested the associations between approximately 10 million genetic variants and circulating levels of 2,994 proteins measured in 3,301 individuals of European descent (30). The association data used in the pQTL analysis were downloaded at http://www.phpc.cam.ac.uk/ceu/proteins/.

### Functional enrichment analysis

To shed light on the pathological significance of the identified proteins in CAD, functional enrichment analyses were performed using the DAVID database (Database for Annotation, Visualization and Integrated Discovery, https://david.ncifcrf.gov/). GO and KEGG pathway analyses were performed to cluster functional genes into different biological processes in which the genes coding the identified proteins were involved. DAVID contains a set of integrated biological knowledge bases and analytic tools for extracting biological significance from large gene/protein lists in a systematic way (31, 32). The results of the functional enrichment analyses are presented in bubble diagrams, which were created by using the “ggplot2” R package.

### MR analysis of proteins

Finally, we attempted to prove that the proteins identified in pQTL analysis were associated with CAD. Five MR methods, including the weighted median (33), inverse-variance weighted (IVW) (34), MR-Egger (35), MR-PRESSO (MR pleiotropy residual sum and outlier) (36) and CAUSE (Causal Analysis Using Summary Effect estimates) (37), were employed to examine the causal associations between circulating protein levels and CAD. In a fixed-effects meta-analysis model, the IVW method combines the ratios of SNP-exposure to SNP-outcome estimates from each instrument variable (34). If more than 50% of the weights for the SNPs come from valid SNPs, the weighted median estimation can produce a consistent assessment (33). The intercept of the MR-egger further tested horizontal pleiotropy (35). The weighted median, IVW and MR-Egger analyses were performed by applying the “mr_allmethods” function in the “MendelianRandomization” R package (38). MR-PRESSO is a method that systematically detects and corrects horizontal pleiotropic outliers in MR testing through three steps: the MR-PRESSO global test, the MR-PRESSO outlier test and the MR-PRESSO distortion test. The outlying genetic variants were identified by applying this method (36). The source code and documents for MR-PRESSO are available at https://github.com/rondolab/MR-PRESSO. The default parameters were used for the MR-PRESSO analysis. The parameters were left to default in the MR-PRESSO analysis.

The requisite data (i.e., SNP rs number, beta, standard error, and *P* value) were extracted from each of the CAD GWASs and pQTL studies mentioned above and then merged by SNP to form a plain file with 7 columns (i.e., SNP rs number, beta for protein, standard error for protein, *P* value for protein, beta for CAD, standard error for CAD and *P* value for CAD) for the MR analysis using the R language. We sorted out the pQTLs with *P* < 5.0 × 10^−6^ as potential instrumental variables. The selection criterion was set to 5.0 × 10^−6^ because 5.0 × 10^−8^ would lead to too few instrumental variables. We harmonized the genetic association between the pQTL and CAD GWAS to ensure that they reflected the same effect allele. We then conducted linkage disequilibrium clumping on these SNPs to obtain the independent pQTL (LD *r*^2^ <0.001, within 10,000 kb) for each protein. Linkage disequilibrium clumping was realized through the “clump_data” function provided by the “TwoSampleMR” R package with reference to the 1000 Genomes EUR population.

We finally applied the CAUSE (an R package) method to differentiate correlated pleiotropy from causal effects for proteins that passed the four MR tests described above. The CAUSE method accounts for both correlated and uncorrelated horizontal pleiotropic effects using summary statistics of genome-wide SNPs (37). CAUSE included as much information from all of the genome-wide variants as possible, even weakly associated variants. Genome-wide summary statistics from the pQTL study and GWASs described above were used in CAUSE analysis. In the analyses, nuisance parameters were estimated using 1,000,000 SNPs. Other parameters were left as the defaults.

## Results

### CAD-associated RNAm-SNPs

We selected RNAm-SNPs from the CAD GWAS datasets according to the annotation information of the RNAm-SNPs. We found 37 RNAm-SNPs that were associated with CAD at *P* < 1.0 × 10^−4^, including 34 m^6^A-, 1 m^1^A- and 2 m^7^G-related SNPs. These 37 RNAm-SNPs mapped to 32 protein coding and noncoding genes.

**Figure 1:**
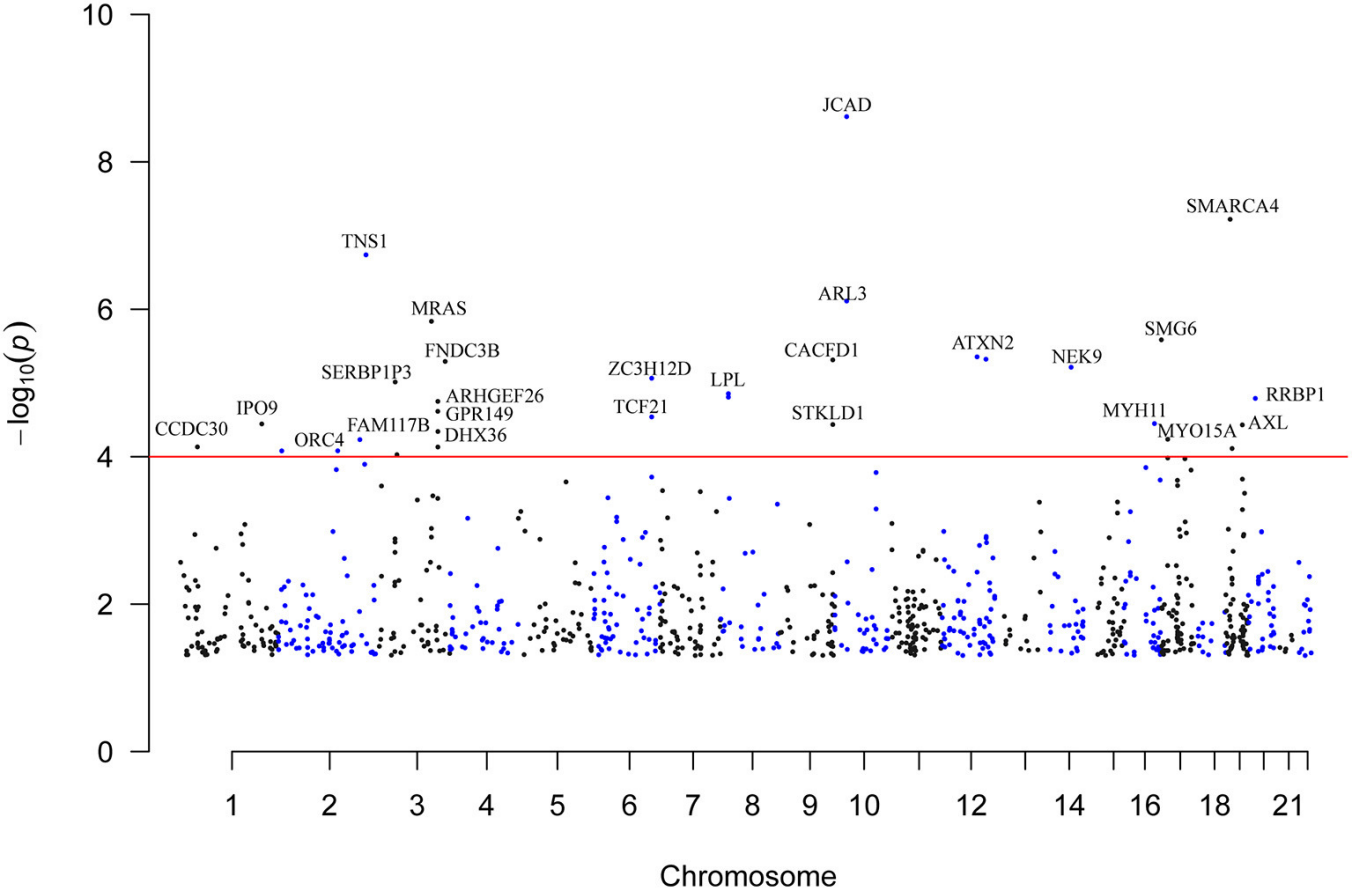
Genome-wide distribution of the identified CAD-associated m^6^A-SNPs. This is a Manhattan plot that shows the *P* values of associations between m^6^A-SNPs and CAD. The x-axis is chromosome positions. The y-axis is the -log_10_ *P* values of the associations. The *P* value information was obtained from the summary dataset of the CAD GWAS. The red line indicates the significance level of 1.0 × 10^−4^.

**Figure 2:**
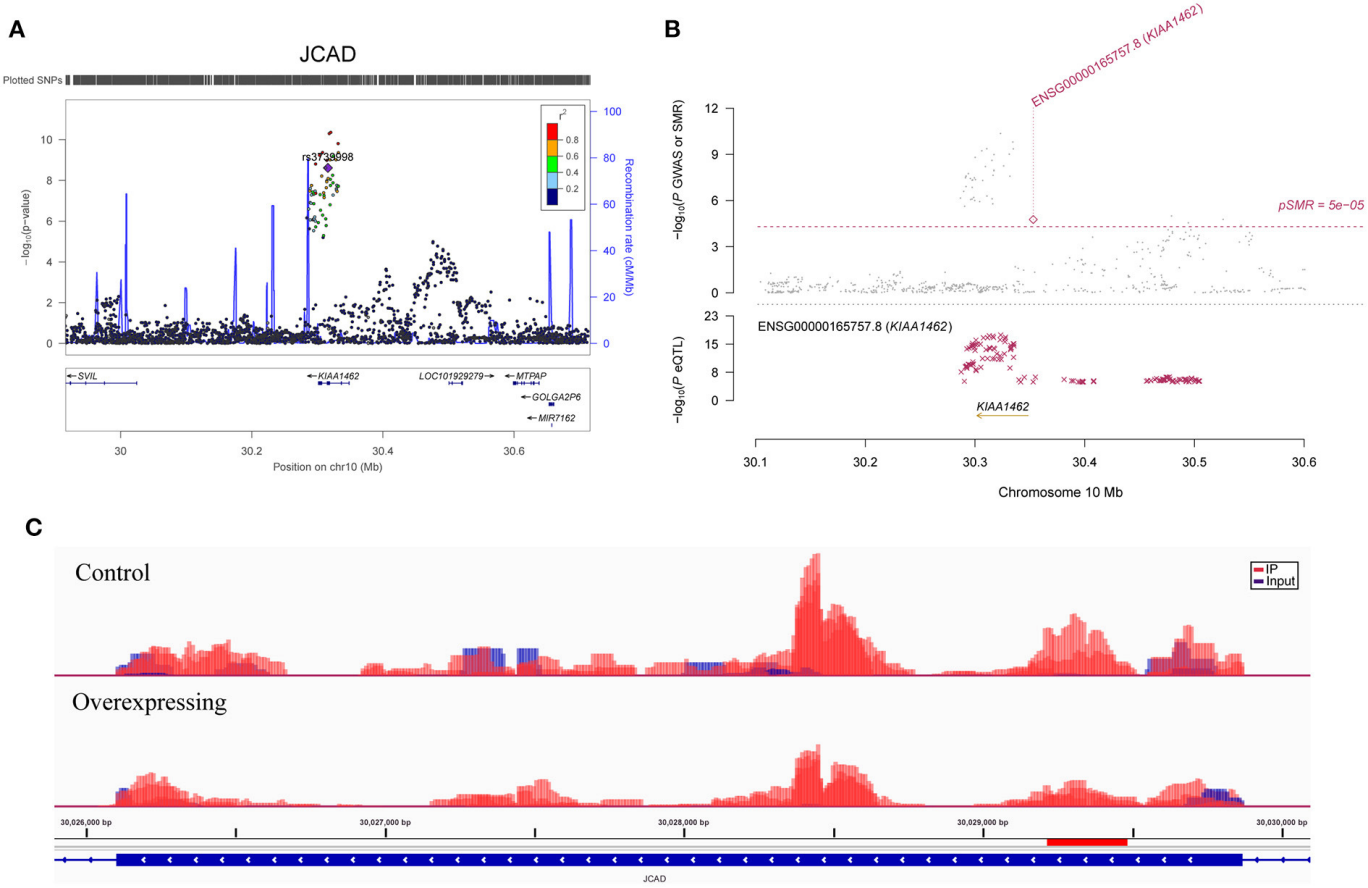
Association between the *JCAD* gene and CAD. **(A)** The m^6^A-SNP rs3739998 in the *JCAD* (*KIAA1462*, reference assembly: GRCh37.p13) gene was associated with CAD; **(B)** SNPs in *JCAD* were strongly associated with the expression level of *JCAD* in aortic artery tissue, and the expression level of *JCAD* in aortic artery tissue was associated with CAD (reference assembly: GRCh37.p13); **(C)** The m^6^A methylation peaks in the sixth exon of *JCAD* in FTO-overexpressing and control HASMCs (reference assembly: GRCh38.p14).

**Figure 3:**
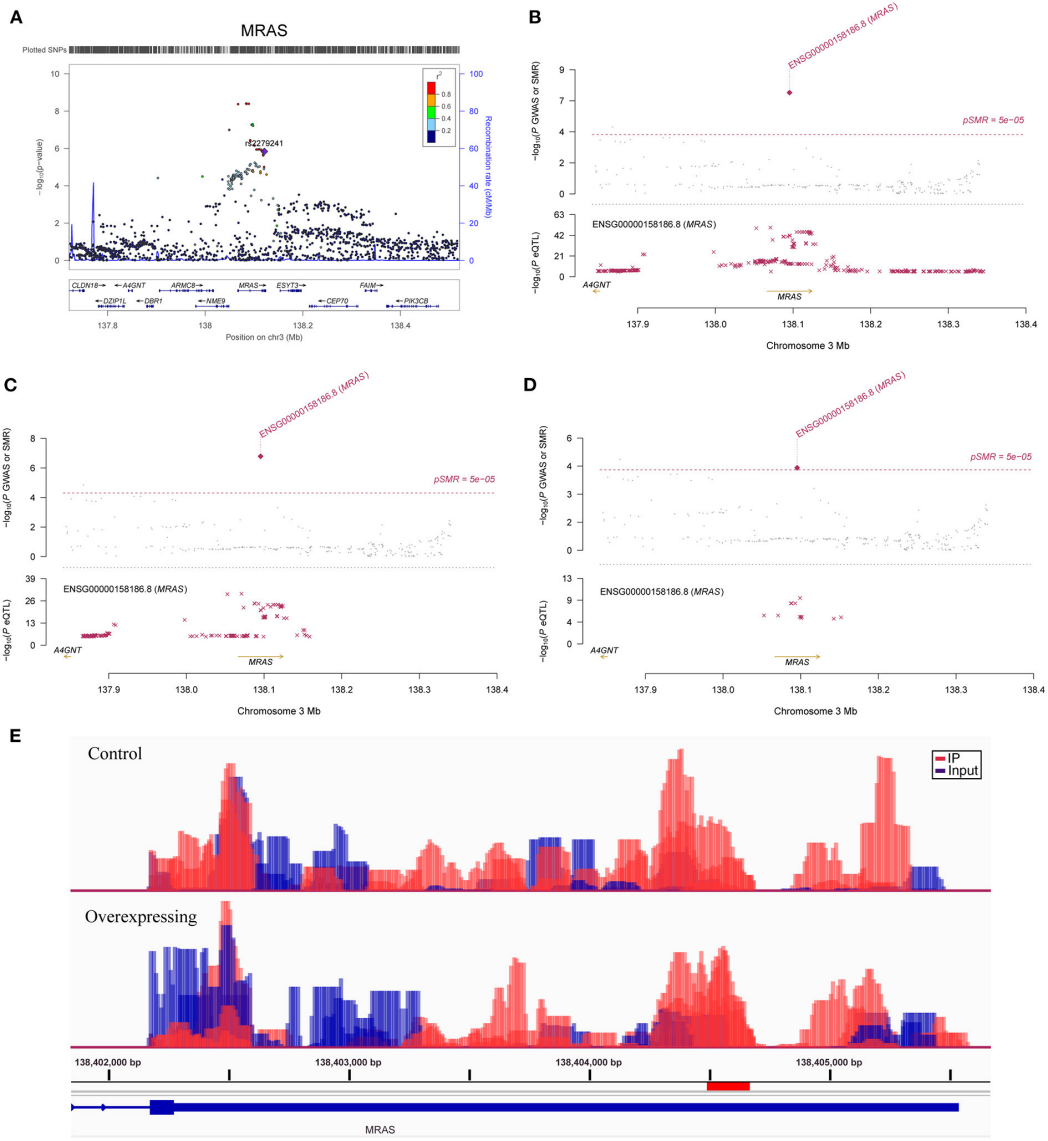
Association between the *MRAS* gene and CAD. **(A)** The m^6^A-SNP rs2279241 (reference assembly: GRCh37.p13) in the *MRAS* gene was associated with CAD; SNPs in *MRAS* were strongly associated with the expression level of *MRAS*, and the expression levels of the *MRAS* gene in aortic artery **(B)**, tibial artery **(C)** and coronary artery tissues **(D)** were associated with CAD (reference assembly: GRCh37.p13); **(E)** The m^6^A methylation peaks in the 3'-UTR of *MRAS* in FTO-overexpressing and control HASMCs (reference assembly: GRCh38.p14).

A total of 923 m^6^A-SNPs among the 9.5 million SNPs were associated with CAD at *P* < 0.05 (Figure 1). Among them, 34 m^6^A-SNPs, including 10 high, 12 medium and 12 low (prediction) confidence levels, were associated with CAD at *P* < 1.0 × 10^−4^. Twenty-seven (79.4%) of them were functional loss, while 7 (20.6%) were functional gain m^6^A-SNPs. One m^6^A-SNP, rs1811351, mapped to the pseudogene SERBP1P3, and the remaining 33 m^6^A-SNPs mapped to 29 protein-coding genes. Of these 33 m^6^A-SNPs mapped to protein coding genes, 10 (30.3%) were exonic, 9 (27.3%) were in the 3'-UTR, 2 (6.1%) were in the 5'-UTR and 12 (36.4%) were intronic. Of note, the m^6^A-SNP rs3739998 (missense variant) at the sixth exon of *JCAD* (*KIAA1462*) was significantly associated with CAD at the genome-wide level (*P* = 2.44 × 10^−9^). This SNP is a m^6^A functional loss and belongs to the medium confidence category. We noticed that rs3739998 is not the top SNP in this locus (Figure 2A). In addition, we also found m^6^A-SNPs in other well-characterized CAD susceptibility genes, such as *MRAS* (Figure 3A), *LPL, TCF21, MYH11* and *SMG6*.

For m^7^G modification, we identified nine m^7^G-SNPs to be nominally associated with CAD. The functional loss m^7^G-associated SNPs rs2270576 (*P* = 9.74 × 10^−6^) in *SNF8* and rs8354 (*P* = 3.01 × 10^−5^) in the 3'-UTR of *ARL3* were associated with CAD. In addition, we found one functional loss m^1^A-SNP rs897172 at the 5'-UTR of *ORC4* that was significantly associated with CAD (*P* = 8.33 × 10^−5^).

### Association with myocardial infarction

Furthermore, we tested for an association between the RNAm-SNPs and AMI in the UK Biobank GWAS. We found 49 RNAm-SNPs that were associated with AMI at *P* < 1.0 × 10^−4^, including 45 m^6^A-, 1 m^5^C-, 1 A-to-I- and 2 m^7^G-related SNPs. The 45 m^6^A-SNPs, including 10 high, 15 medium and 20 low (prediction) confidence levels, mapped to 6 noncoding genes and 38 protein coding genes. Among the 39 m^6^A-SNPs mapped to protein coding genes, 8 (20.5%) were exonic, 13 (33.3%) were in the 3'-UTR, 2 (5.1%) were in the 5'-UTR and 16 (41.0%) were intronic. Three m^6^A-SNPs, rs764957 in LOC100506178, rs148172130 in *RPL14* and rs12190287 in *TCF21*, were significantly associated with AMI at the genome-wide level (*P* = 3.95 × 10^−10^, 2.46 × 10^−8^ and 3.95 × 10^−8^, respectively). We found that a missense variant rs72654423 (m^6^A-SNP) in *APOB* was nominally associated with AMI (*P* = 7.27 × 10^−5^). The m^7^G-SNP rs186643756 in the lncRNA *PVT1* was significantly associated with CAD at the genome-wide level (*P* = 3.20 × 10^−8^). For the 37 CAD-associated RNAm-SNPs described above, we found four m^6^A-SNPs (rs13702 and rs3208305 in the 3'-UTR of *LPL*, rs12190287 in the 3'-UTR of *TCF21* and rs6490162 in the intron of *ATXN2*) that were associated with AMI (*P* < 1.0 × 10^−4^). Taken together, we identified 81 RNAm-SNPs (three types of modification) associated with CAD or AMI at *P* < 1.0 × 10^−4^ (Supplementary Table S1).

### Gene expression related to CAD

We further investigated whether the 81 identified RNAm-SNPs were associated with the expression levels of their host genes. Many RNAm-SNPs displayed eQTL effects with the corresponding genes in various types of tissues according to the HaploReg database. Thirty-six eQTLs were found, and 28 of them were significantly associated with the expression of their host genes (cis-eQTLs), including *LPL, TCF21, MRAS, JCAD, ATXN2, MAPKAPK5, MYH11, SNF8, MYO15A, IPO9, ORC4, TNS1, IP6K2, SERBP1P3, ARHGEF26, DHX36, PARP12, CACFD1, ARL3, POC1B, NEK9, CFDP1, ZNF652, SMARCA4, CYP4F2* and *RRBP1*. Specifically, rs2279241 in *MRAS* was associated with the expression levels of *MRAS* in subcutaneous adipose, aortic artery and tibial artery tissues; rs12190287 in *TCF21* was associated with the expression levels of *TCF21* in subcutaneous adipose tissue; rs13702 in *LPL* was associated with the expression levels of *LPL* in whole blood cells; rs6490162 in *ATXN2* was associated with the expression levels of *ATXN2* in whole blood cells and was also associated with the expression levels of *SH2B3* in lymphoblastoid and whole blood cells; rs2075511 in *MYH11* was associated with the expression levels of *MYH11* in whole blood cells; rs2270576 in *SNF8* was associated with the expression levels of *SNF8* in aortic artery and adrenal gland and was associated with the expression levels of *UBE2Z* and *ATP5G1* in whole blood cells; and two missense variants, rs3739998 and rs2185724, in *JCAD* were associated with the expression levels of *JCAD* in the aortic artery. In addition, in skeletal muscle and the tibial artery, rs854766 in the intron of *MYO15A* was strongly associated with the expression of *ALKBH5*, which is a m^6^A demethylase.

In SMR analysis, we found significant associations between gene expression levels in the 11 relevant tissue types and CAD. We set the significance threshold to 5.0 × 10^−5^ and detected significant associations for *FAM117B, MRAS, DHX36, TCF21, JCAD, ARL3, SH2B3, MAPKAPK5, CFDP1, ATP5G1, SMARCA4* and *RRBP1* (Supplementary Table S2). We found significant associations between the expression levels of *JCAD* in the aortic artery (*P* = 1.73 × 10^−5^) (Figure 2B), *MRAS* in the aortic artery (*P* =4.59 × 10^−8^) (Figure 3B), tibial artery (*P* =1.64 × 10^−7^) (Figure 3C), coronary artery (*P* = 3.89 × 10^−5^) (Figure 3D) and atrial appendage (*P* = 1.43 × 10^−5^), *FAM117B* in the aortic artery (*P* =4.78 × 10^−6^), *DHX36* in whole blood cells (*P* = 2.02 × 10^−5^), *SH2B3* in whole blood cells (*P* = 2.04 × 10^−5^), *ATP5G1* in whole blood cells (*P* =3.03 × 10^−6^), left ventricle (*P* =7.32 × 10^−6^) and atrial appendage (*P* = 1.66 × 10^−5^), *SMARCA4* in whole blood cells (*P* = 4.82 × 10^−5^), *RRBP1* in whole blood cells (*P* = 2.05 × 10^−5^) and CAD. In addition, suggestive associations were found between the expression levels of *DHX36* in whole blood cells (*P* = 5.96 × 10^−5^), *TCF21* in the tibial artery (*P* = 8.85 × 10^−5^) and subcutaneous adipose tissue (*P* = 7.76 × 10^−5^), *ARL3* in whole blood cells (*P* = 8.64 × 10^−5^), *MAPKAPK5* in whole blood cells (*P* = 7.44 × 10^−5^), and *CFDP1* in whole blood cells (*P* = 8.71 × 10^−5^) and CAD.

### Differentially methylated and expressed RNAs in FTO-overexpressing HASMCs

In FTO-overexpressing HASMCs, we aimed to determine which of the CAD loci contain methylation sites, whether the expression of the methylated genes is affected, and which of the CAD-associated m^6^A-SNPs fall into the differentially methylated peaks. For the genes containing m^6^A-SNPs, we found that 19 were differentially methylated in FTO-overexpressing HASMCs (fold change > 2.0, FDR <0.05) (Supplementary Table S3). We noticed that key CAD genes, such as *JCAD* (Figure 2C), *MRAS* (Figure 3E), *MYH11* and *SMG6*, were differentially methylated, and *SMG6* was differentially expressed in FTO-overexpressing HASMCs. We found that the synonymous m^6^A-SNP rs216196 (chr17: 2299651) was located in differentially methylated peaks (chr17: 2299633 - 2299784) in the *SMG6* genes. In addition, we found that *UBE2Z* and *SH2B3* were differentially methylated and differentially expressed in FTO-overexpressing HASMCs. For the differentially methylated genes, the expression levels of *MAPKAPK5, MRAS* and *SH2B3* in the aorta, coronary artery, tibial artery or whole blood cells were associated with CAD in the SMR analysis described above (Supplementary Table S2).

### Differentially methylated and expressed RNAs in PBMCs

Among the genes that contain the RNAm-SNPs identified above, we further found that *ASAP2, MRAS, ARHGEF26, MDN1, MTHFSD, SMG6, MYO15A, OSBPL7, AIRE, FTCD* and *MAPK11* were differentially methylated (seven upregulated and four downregulated; fold change > 2.0, FDR <0.05) in human PBMCs of five CAD cases and five controls (Supplementary Table S4). In human PBMCs of 93 CAD cases and 48 controls, 42 genes seemed to be differentially expressed (FDR <0.05), including *APOB, ATXN2, MYH11, UBE2Z, ATP5G1* and *SH2B3* (Supplementary Table S5). However, only *APOB* passed the significance threshold of fold change > 2.0, FDR <0.05 (fold change = 2.0, FDR = 3.73 × 10^−5^).

### Proteins affected by the RNAm-SNPs

We identified 65 pQTL signals (*P* < 5.0 × 10${}_{,}^{-8}$ Supplementary Table S6) for six of the identified RNAm-SNPs (rs739468, rs41302673, rs6859, rs3172494, rs11105310 and rs8354). A total of 44 proteins were identified. rs739468 in *CACFD1* was associated with circulating levels of 26 proteins; rs41302673 in *STKLD1* was associated with circulating levels of 20 proteins. The top signals were the significant associations between rs739468 in *CACFD1* and circulating levels of SELE (*P* = 2.04 × 10^−134^) and ABO (*P* = 3.80 × 10^−126^). rs41302673 in *STKLD1* was also associated with circulating levels of SELE (*P* = 1.0 × 10^−89^) and ABO (*P* = 3.16 × 10^−84^). The 3'-UTR SNP rs8354 in *ARL3* was significantly associated with ARL3 levels (*P* = 2.57 × 10^−8^).

**Figure 4:**
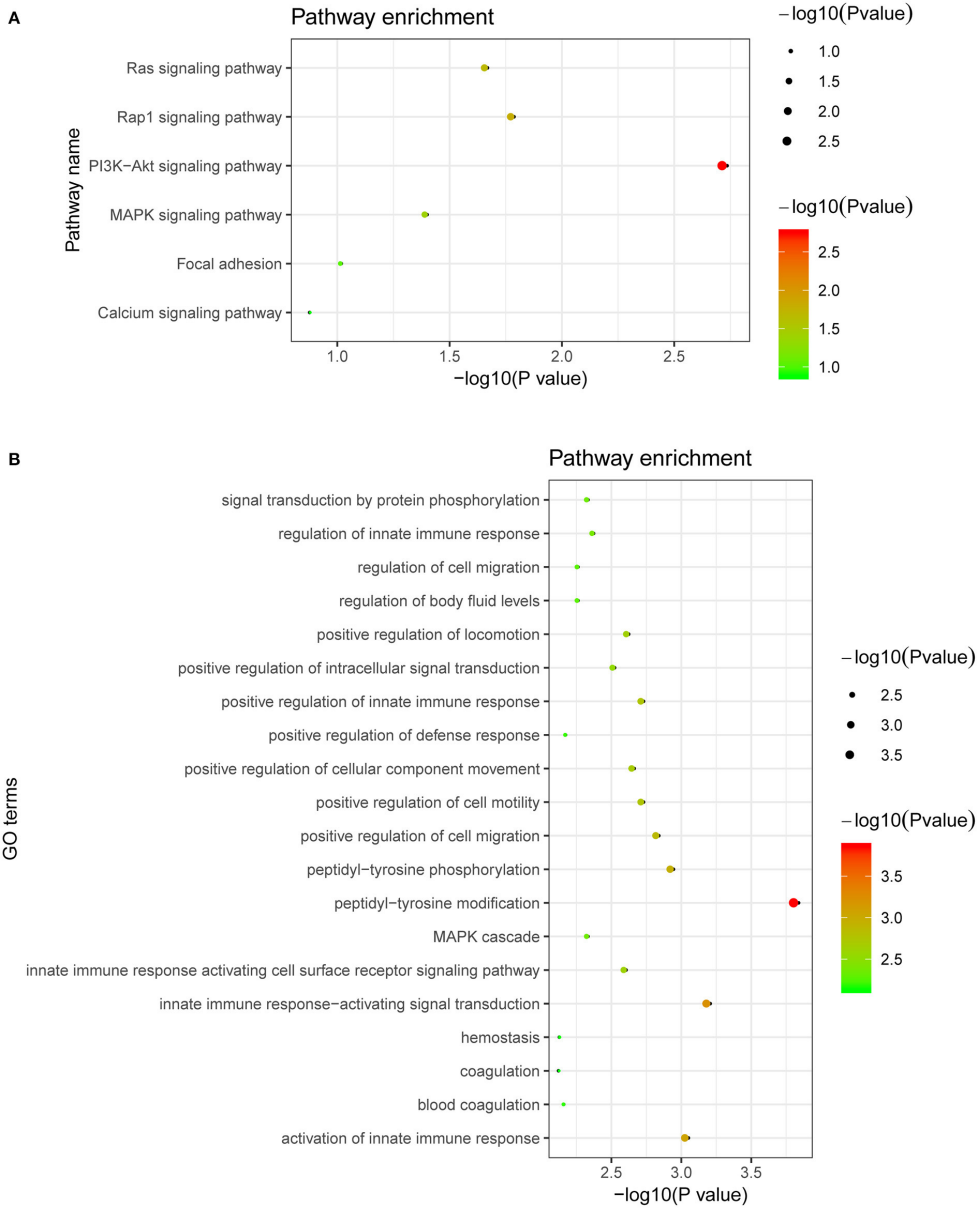
Biological pathways related to the proteins affected by the CAD-associated RNAm-SNPs. **(A)** KEGG pathway enrichment of the proteins affected by the CAD-associated RNAm-SNPs; **(B)** The top 20 significant biological process GO terms for the proteins affected by the CAD-associated RNAm-SNPs.

We performed GO and KEGG pathway analyses in the DAVID database for proteins that were affected by the CAD-associated RNAm-SNPs. We mainly analyzed the 44 proteins related to the RNAm-SNPs. The identified proteins (FLT4, INSR, IL3RA, KDR, MET and TLR4) were enriched in the PI3K-Akt signaling pathway (*P* = 1.80 × 10^−3^), Rap1 signaling pathway (*P* = 1.60 × 10^−2^), Ras signaling pathway (*P* = 2.10 × 10^−2^) and MAPK signaling pathway (*P* = 3.90 × 10^−2^) (Figure 4A). The 44 associated proteins were also enriched in 58 biological process GO terms, including peptidyl-tyrosine modification (TPST2, CTF1, CD300A, FLT4, INSR, KDR and MET, *P* = 1.40 × 10^−4^), innate immune response-activating signal transduction (CD300A, CD209, PSME1, ICAM2, TLR4 and MBL2, *P* = 6.0 × 10^−4^), positive regulation of cell migration (SELP, FLT4, INSR, KDR, SELE, MET and TLR4, *P* = 1.40 × 10^−3^), MAPK cascade (CD300A, FLT4, INSR, IL3RA, PSME1, KDR, MET and TLR4, *P* = 4.50 × 10^−3^), blood coagulation (SELP, F8, C1GALT1C1, DOCK9 and TLR4, *P* = 6.70 × 10^−3^) and so on (Figure 4B).

### Proteins causally associated with CAD

We tested whether circulating levels of these 44 proteins were causally associated with CAD using five MR methods to support the role of the RNAm-SNPs in CAD. Associations with *P* < 1.14 × 10^−3^ were considered significant in this analysis. We found that the associations between circulating levels of ABO, C1GALT1C1, C5orf38, CD209, CEP57, CTF1, F8, GNAI3, GOLM1, IL3RA, LRRN1, PSME1, QSOX2, SELE, VIMP and VPS29 and CAD were significant in weighted median, IVW, MR-Egger and MR-PRESSO analyses (Table 1). Proteins F8, C1GALT1C1 and GNAI3 were enriched in the blood coagulation biological process (*P* = 2.30 × 10^−2^). We further examined the potential causal associations between these 16 proteins and AMI. The associations between circulating levels of 12 proteins, including C1GALT1C1, C5orf38, CD209, CEP57, CTF1, F8, GNAI3, LRRN1, PSME1, QSOX2, VIMP and VPS29, and AMI were significant in weighted median, IVW, MR-Egger or MR-PRESSO analyses (Table 1).Therefore, the associations between circulating levels of these 12 proteins (including the three proteins enriched in blood coagulation biological process) and CAD were strengthened.

**Table 1:** Association between circulating protein levels and CAD and AMI.

**Proteins**	**Estimate ¶**	**Standard Error ¶**	***P*** **values**					
			**MR-PRESSO**	**IVW**	**Weighted median**	**MR-Egger**	**Intercept**	**CAUSE**
**CAD**								
ABO	0.0363	0.0049	2.43E-09	2.29E-13	3.03E-06	9.42E-08	5.66E-01	9.10E-03
C1GALT1C1	0.0737	0.0157	5.33E-05	2.84E-06	4.27E-07	8.13E-07	5.94E-03	9.69E-02
C5orf38	0.0857	0.0125	6.75E-09	7.85E-12	4.22E-18	7.18E-09	8.23E-02	7.89E-03
CD209	0.0452	0.0089	9.30E-06	3.86E-07	5.73E-06	6.92E-05	2.54E-01	1.64E-02
CEP57	−0.0681	0.0120	6.59E-08	1.50E-08	2.52E-11	9.14E-08	3.14E-02	4.27E-02
CTF1	0.0965	0.0118	1.64E-09	3.48E-16	2.75E-13	6.44E-15	2.41E-03	1.10E-04
F8	0.0591	0.0145	6.17E-04	4.72E-05	1.28E-04	1.07E-06	2.39E-03	5.29E-02
GNAI3	−0.0741	0.0207	1.38E-03	3.43E-04	3.91E-04	8.83E-05	2.52E-02	8.34E-02
GOLM1	0.0618	0.0153	3.31E-04	5.07E-05	1.27E-08	1.11E-04	4.17E-02	1.82E-01
IL3RA	−0.0549	0.0095	1.02E-06	7.16E-09	6.48E-08	2.61E-08	1.38E-02	3.93E-02
LRRN1	0.0689	0.0102	1.63E-07	3.03E-09	2.29E-10	1.59E-06	2.54E-01	9.43E-03
PSME1	−0.0771	0.0133	3.24E-06	3.96E-07	3.01E-08	2.65E-08	4.53E-03	5.48E-02
QSOX2	0.0567	0.0079	5.62E-09	7.25E-13	1.34E-10	2.14E-13	8.38E-04	1.01E-02
SELE	−0.0663	0.0069	1.49E-11	4.29E-06	4.90E-13	9.32E-07	2.16E-02	4.19E-03
VIMP	0.0860	0.0143	9.38E-07	7.65E-11	1.66E-14	2.45E-13	2.00E-04	1.34E-02
VPS29	−0.0640	0.0104	2.88E-07	3.22E-08	6.42E-12	1.00E-08	9.47E-03	6.06E-02
**AMI**								
ABO	0.0005	0.0003	1.10E-01	1.37E-01	7.38E-02	3.98E-01	9.90E-01	1.20E-01
C1GALT1C1	0.0010	0.0004	2.62E-02	1.84E-02	4.65E-04	8.71E-03	1.20E-01	1.41E-01
C5orf38	0.0020	0.0004	1.39E-04	2.66E-06	4.57E-08	3.71E-08	1.45E-02	1.20E-02
CD209	0.0013	0.0003	2.10E-03	1.17E-04	1.89E-04	1.42E-03	3.28E-01	4.24E-02
CEP57	−0.0013	0.0004	6.95E-03	1.97E-03	5.84E-08	4.91E-07	1.40E-03	3.96E-02
CTF1	0.0023	0.0004	2.32E-04	2.33E-08	7.84E-11	1.10E-09	9.91E-03	8.09E-03
F8	0.0018	0.0004	1.40E-03	1.56E-04	3.41E-04	1.73E-01	6.41E-01	1.75E-02
GNAI3	−0.0017	0.0006	3.31E-03	6.96E-03	3.69E-05	9.72E-04	5.20E-02	1.54E-01
GOLM1	0.0013	0.0006	3.59E-02	4.30E-01	3.73E-02	1.26E-01	1.94E-01	9.99E-01
IL3RA	−0.0011	0.0005	6.41E-02	4.44E-02	4.65E-03	2.47E-01	8.60E-01	9.20E-01
LRRN1	0.0022	0.0003	5.35E-06	4.72E-11	8.07E-11	3.13E-08	2.11E-01	2.12E-02
PSME1	−0.0017	0.0004	1.81E-03	1.24E-04	1.40E-05	1.49E-07	2.04E-03	2.54E-01
QSOX2	0.0011	0.0004	9.03E-03	2.18E-03	1.67E-04	6.35E-04	6.16E-02	5.14E-02
SELE	−0.0006	0.0005	2.10E-01	1.92E-01	2.24E-03	2.02E-03	6.58E-03	2.05E-01
VIMP	0.0021	0.0005	1.10E-03	6.46E-05	2.02E-08	6.07E-04	3.05E-01	8.41E-03
VPS29	−0.0014	0.0003	9.76E-05	2.66E-07	1.20E-08	7.58E-08	3.83E-02	1.11E-01

¶: The effect estimation was derived from the MR-PRESSO analysis.

**Figure 5:**
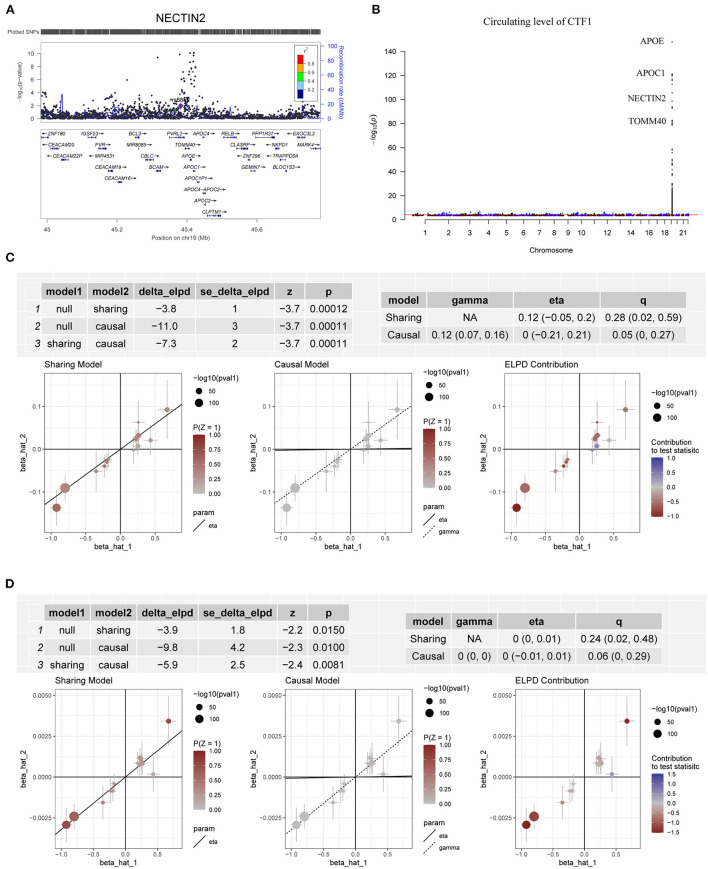
Association between circulating levels of CTF1 and CAD. **(A)** The m^6^A-SNP rs6859 in the *NECTIN2* gene (*PVRL2*, in 19q13.32, reference assembly: GRCh37.p13) was associated with CAD; **(B)** A total of 263 SNPs in 19q13.32 were significantly (*P* < 5.0 × 10^−8^) associated with the circulating level of CTF1; **(C)** The association between CTF1 level and CAD in the CAUSE analysis. The causal model was significantly better than both the null and the sharing models; **(D)** The association between CTF1 level and AMI in the CAUSE analysis. The causal model was significantly better than both the null and the sharing models.

In the CAUSE analysis, which accounted for correlated and uncorrelated horizontal pleiotropic effects, circulating levels of ABO, C5orf38, CD209, CEP57, CTF1, IL3RA, LRRN1, QSOX2, SELE and VIMP were associated with CAD; C5orf38, CD209, CEP57, CTF1, F8, LRRN1 and VIMP levels were found to be associated with AMI (Table 1). The most significant protein was CTF1 (cardiotrophin-1), which was associated with both CAD (*P* = 1.10 × 10^−4^) and AMI (*P* = 8.09 × 10^−3^) in the CAUSE analysis (Figure 5).

## Discussion

In the present study, we integrated information from the RMVar database, CAD GWAS and QTL studies to identify CAD-associated RNAm-SNPs related to m^6^A, m^1^A, m^5^C, m^7^G and A-to-I modification types. Some of the RNAm-SNPs were associated with the expression of the local genes in CAD-relevant tissues, and the genes were differentially expressed in CAD. We also found some of the RNAm-SNPs to be associated with circulating protein levels, and the related proteins were associated with CAD and AMI. This study highlighted the importance of detecting RNAm-SNPs in CAD susceptibility genes by excavating the conveniently available SNP association data of GWAS, and the findings indicated that RNAm-SNPs might be potential functional variants for CAD.

Hundreds of genomic loci associated with CAD have been identified in GWASs. However, the identification of functional variants, causal gene(s) or protein(s) remains a major challenge. The role of m^6^A methylation in CAD has been shown (24). Some genetic variants in CAD loci may alter m^6^A methylation and then disturb gene expression regulation (39). Recently, a study integrating m^6^A QTLs with disease genetics identified 184 GWAS-colocalized m^6^A QTLs, including 50 muscle/heart m^6^A QTLs underlying CAD (40). Until now, the functional roles and underlying molecular mechanisms of RNA modification in CAD remained largely unclear. In the present study, we identified many RNAm-SNPs in CAD susceptibility genes and showed that the RNAm-SNPs may have functional roles in gene expression at the mRNA and protein levels. In FTO-overexpressing HASMCs, we showed that CAD susceptibility genes were differentially methylated and that some of the m^6^A-SNPs were located in a differentially methylated peak (e.g., rs216196 in *SMG6*). Differential methylation and expression of CAD susceptibility genes between CAD cases and controls were also found in PBMCs. These findings suggest that RNA modification may play critical roles in the pathogenesis of CAD.

We found several RNAm-SNPs that were associated with gene expression, and the gene expression levels were associated with CAD. The m^6^A-SNP rs3739998 was associated with *JCAD* expression in aortic artery tissue, and the expression levels of *JCAD* in the aortic artery were associated with CAD. *JCAD* (Junctional cadherin 5 associated, also known as *KIAA1462*), which encodes the junctional protein associated with CAD, is one of most important GWAS-identified genes for CAD. Genetic variants in the *JCAD* gene region were significantly associated with CAD and AM (7, 8, 41). A study (42) showed an association of this nonsynonymous SNP with CAD in the German MI Family Study with a combined *P* = 1.27 × 10^−11^. Furthermore, Xu et al. (43) demonstrated that *JCAD* depletion in endothelial cells inhibited the activation of the YAP/TAZ pathway and the expression of downstream proatherogenic genes. They also showed that *JCAD* regulates YAP/TAZ protein activation by interacting with TRIOBP, an action-binding protein. Therefore, *JCAD* stabilizes stress fiber formation and thereby plays a functional role in the pathogenesis of CAD. Our study highlighted rs3739998, which may affect *JCAD* expression, as a potential functional variant for CAD and suggested the possibility of attractive new therapeutic strategies for CAD by targeting *JCAD*.

To avoid a large number of false positive results, previous GWASs used strict significance thresholds. However, due to the use of strict significance levels, many moderate association signals in the GWAS dataset were ignored. In addition to the genome-wide significant SNP in *JCAD*, RNAm-SNPs in other important CAD susceptibility genes were also identified, including *MRAS* and *TCF21*. These RNAm-SNPs are not genome-wide significant, but they significantly affected the expression levels of *MRAS* and *TCF21*, and the expression levels of *MRAS* and *TCF21* were genetically associated with CAD. *DHX36*, which is involved in the regulation of cardioblast differentiation and proliferation during heart development and plays a role in many biological processes, such as genomic integrity (44), transcriptional regulation (45, 46) and posttranscriptional regulation (47), is another example. We found that four m^6^A-SNPs in 3q25.2, rs12493885 and rs357504 in *ARHGEF26*, rs403132 in *DHX36* and rs701133 in *GPR149*, were associated with the expression levels of *DHX36*. The associations between these RNAm-SNPs and CAD are not genome-wide significant. These variants are proximal to previously described loci associated with CAD, specifically the 3'-UTR variant rs701145 (10) and the intron variants rs1727949 (48) and rs789294 (10). *DHX36* expression levels in blood cells were found to be genetically associated with CAD in our SMR analysis. Hence, it is possible that RNA methylation may affect *DHX36* gene expression and then have a pathogenic effect on CAD. Overall, these findings suggested that the RNAm-SNPs may affect CAD risk by affecting gene expression and therefore may be functional variants in the CAD loci.

Circulating proteins are druggable targets (49, 50). A great number of studies have been conducted to identify circulation proteins contributing to CAD. However, traditional observational studies have limitations in disentangling which factors causally affect CAD. MR-based studies can address these limitations by use of genetic proxies of putative risk factors when evaluating their associations with disease risk, as they are not subject to reverse causation (51). More importantly, the MR method allows the evaluation of the association between gene expression levels and CAD risk in very large samples by using data from large-scale GWAS. Some of the new MR methods, such as CAUSE, avoid more false positives induced by correlated and uncorrelated horizontal pleiotropic effects (37). As we showed in this study, MR analysis provided robust evidence of the vital roles of several proteins in CAD.

According to the pQTL analysis, we found that the RNAm-SNPs were associated with circulating levels of many proteins, including proteins involved in the Rap1 signaling pathway, Ras signaling pathway, PI3K-Akt signaling pathway and MAPK signaling pathway. Activation of the PI3K/Akt signaling pathway is well known to accelerate the development of atherosclerosis (52). As suggested by a recently published study (53), this pathway plays an important role in the pathology of CAD. The affected proteins play functional roles in biological processes of peptidyl-tyrosine modification, innate immune response-activating signal transduction, positive regulation of cell migration, MAPK cascade, blood coagulation and so on. Afterward, by applying several MR methods, we showed that the proteins affected by the RNAm-SNPs were causally associated with CAD, including proteins function in the blood coagulation process (F8, C1GALT1C1 and GNAI3). The strongest evidence was found for cardiotrophin-1, which was significantly associated with both CAD and AMI in all MR analyses using five methods. The associations between SNPs in 19q13.32 (*APOE, APOC1, NECTIN2, TOMM40, BCAM, CEACAM16, CBLC, APOC4, BCL3, RELB, CLPTM1, NKPD1, CEACAM19, PVR, IGSF23, CLASRP, TRAPPC6A* and *PPP1R37*) and CAD and circulating levels of CTF1 contributed to the identification of the causal association between circulating levels of CTF1 and CAD. CTF1 is a procardiogenic factor that has been shown to play important roles in cardiovascular disease (54–56). Identification of this key protein provided a better entry point for studies on the pathological mechanism of CAD. Although the m^6^A-SNP rs6859 was found in *NECTIN2*, which encodes nectin cell adhesion molecule 2, functional genetic variants in this locus need to be confirmed in future studies.

Some limitations of the current work need to be acknowledged. First, the data used were mainly from European populations; therefore, the results may need to be extrapolated carefully. Second, although the m^6^A-SNP dataset was extensive, information on other types of RNA modification was still scarce. Third, the sample size of the pQTL study was relatively small, and therefore, the estimations of the effects of proteins on CAD risk may not be so accurate. Finally, this work is based on statistical evidence, and the functions of the identified RNAm-SNPs and genes have not been validated experimentally. Further experimental studies are required to determine their functions.

In conclusion, the present study identified CAD-associated RNAm-SNPs and suggested that RNA modification may play a role in the pathogenesis of CAD. RNAm-SNPs in CAD susceptibility genes may regulate gene expression at the mRNA (e.g., *JCAD, MRAS, TCF21*, and *DHX36*) or protein (e.g., ABO, C5orf38, CD209, CEP57, CTF1, IL3RA, LRRN1, QSOX2, SELE and VIMP) levels. The relationships between RNAm-SNPs, RNA modification, gene expression and CAD have not been clarified in previous studies. Therefore, this study increased our understanding of the genetic association signals identified in the CAD GWAS and identified additional risk factors for CAD.

## Data availability statement

The original contributions presented in the study are included in the article/Supplementary material, further inquiries can be directed to the corresponding authors.

## Ethics statement

The studies involving human participants were reviewed and approved by Soochow University. The patients/participants provided their written informed consent to participate in this study.

## Author contributions

HZ and XM obtained funding, performed the analyses, and rafted the initial manuscript. XM and LW designed the study. CD, RL, FT, DL, and NW were involved in the study design and/or data collection. XM, YZ, and LW have primary responsibility for the final content. XM is the guarantor of this study. All authors read and approved the final manuscript.

## Funding

This study was supported by the Natural Science Foundation of China (82173597, 82170480, 82073636, and 81773508), the CAMS Innovation Fund for Medical Sciences (CIFMS) (2021-I2M-1-008), the Startup Fund from Soochow University (Q413900313, Q413900412), and a Project of the Priority Academic Program Development of Jiangsu Higher Education Institutions.

## Conflict of interest

The authors declare that the research was conducted in the absence of any commercial or financial relationships that could be construed as a potential conflict of interest.

## Publisher's note

All claims expressed in this article are solely those of the authors and do not necessarily represent those of their affiliated organizations, or those of the publisher, the editors and the reviewers. Any product that may be evaluated in this article, or claim that may be made by its manufacturer, is not guaranteed or endorsed by the publisher.
